# Effects of psychological birth trauma on obsessive-compulsive behaviors during the postpartum period in terms of infant care: the chain-mediating role of intolerance of uncertainty and dyadic coping

**DOI:** 10.3389/fpubh.2026.1876448

**Published:** 2026-07-15

**Authors:** Xiaotong Zhang, Qi Xu, Minmin Li, Ye Zhang, Jia Song, Xia Liu

**Affiliations:** 1 School of Nursing, Shandong First Medical University, Taian, China; 2 Department of Neonatology, East Campus, Shandong Provincial Hospital Affiliated to Shandong First Medical University, Jinan, China; 3 Department of Obstetrics, East Campus, Shandong Provincial Hospital Affiliated to Shandong First Medical University, Jinan, China; 4 Department of Nursing, Shandong Provincial Hospital Affiliated to Shandong First Medical University, Jinan, China

**Keywords:** birth trauma, chain mediating effects, infant care, obsessive-compulsive behaviors, obsessive-compulsive disorder

## Abstract

**Background:**

Obsessive-compulsive disorder is one of the common mental health problems among perinatal women, and the postpartum period represents a particularly vulnerable stage for the emergence of clinically significant obsessive-compulsive symptoms. Postpartum infant care–related obsessive-compulsive behaviors may exacerbate maternal negative emotions such as anxiety and depression through a cognitive–behavioral cycle and impair the development of the mother-infant relationship. Birth trauma is one of the influencing factors of perinatal mental health, and obsessive-compulsive behaviors may develop or worsen as a response to birth trauma, highlighting the need to explore the potential psychological mechanisms underlying the association between birth trauma and postpartum infant care-related obsessive-compulsive behaviors. This study examined the relationship between birth trauma and postpartum obsessive-compulsive behaviors, as well as the chain mediating role of intolerance of uncertainty and dyadic coping.

**Methods:**

A total of 278 hospitalized parturients were recruited using the convenience sampling method, and the questionnaires included the general information questionnaire, Psychological Birth Trauma Scale, Intolerance of Uncertainty Scale, Dyadic Coping Scale, and Postpartum Infant Care-Obsessive Compulsive Behavior Scale. Data were analyzed using correlation analysis in SPSS, and the PROCESS macro was employed for mediation effect analysis.

**Results:**

(1) Birth trauma exerted a significant positive effect on postpartum infant care-related obsessive-compulsive behaviors. (2) The association between birth trauma and postpartum obsessive-compulsive behaviors was independently mediated by intolerance of uncertainty. (3) Dyadic coping independently mediated the relationship between birth trauma and postpartum care-related obsessive-compulsive behaviors. (4) Intolerance of uncertainty and dyadic coping played a chain mediating role between birth trauma and postpartum care-related obsessive-compulsive behaviors. Our study enriches existing research on birth trauma and postpartum care-related obsessive-compulsive behaviors, deepens the understanding of their effects, and provides a reference for optimizing perinatal care practice.

**Conclusion:**

Intolerance of uncertainty and dyadic coping serve as important mediators between birth trauma and postpartum infant care-related obsessive-compulsive behaviors, providing key evidence for developing potential interventions and support services in perinatal mental health. Future efforts should focus on maternal childbirth experiences and postpartum negative emotional and behavioral problems, provide sufficient social support, and improve family infant caregiving skills.

## 1 Introduction

Obsessive-compulsive disorder is one of the common psychiatric disorders among perinatal women (1), and the postpartum period represents a stage in which women are particularly vulnerable to the emergence of clinically significant obsessive-compulsive symptoms (2, 3). Approximately 83% of perinatal obsessive-compulsive disorder cases are diagnosed postpartum, and some parturient women who do not meet the criteria for a clinical diagnosis already fulfill the relevant conditions for a subclinical diagnosis (4). Obsessive-compulsive behaviors are regarded as transdiagnostic symptoms, they are a hallmark of numerous psychiatric disorders (5–8), and they can exacerbate the onset and worsening of negative emotions such as maternal anxiety and depression through cognitive–behavioral cycles (9, 10). Obsessive-compulsive behaviors during the postpartum period in terms of baby care are triggered by abrupt and drastic hormonal changes, sleep deprivation, role transition, and negative experiences during pregnancy or childbirth, which heighten the level of maternal sensitivity toward the infant, generate fears regarding potential harm to the baby, and foster an overprotective attitude; mothers with obsessive thoughts display maladaptive care-related obsessive-compulsive behaviors, including insomnia, excessive cleaning or checking of the infant, and controlling tendencies, all of which are intended to prevent feared events and reduce anxiety or distress. The progression and aggravation of such behaviors can impair the maternal caregiving capacity and limit sufficient mother–infant contact (11), mothers with comorbid depression show insensitivity in infant interaction and care, Challacombe et al. (3) and they have reduced breastfeeding self-efficacy (12), which adversely affects mother–infant bonding and may further lead to cognitive and behavioral developmental disturbances in infants (13). Furthermore, the transdiagnostic nature of postpartum obsessive-compulsive behaviors (14) can result in their underdiagnosis or misclassification as post-partum anxiety, depression, or related disorders, and a lack of awareness among caregivers or parturient women can prevent timely access to effective mental health care and parenting guidance (13, 15). In the Guidelines for Maternal and Child Health Services for Perinatal Mental Health, perinatal mental health services are formally integrated into the WHO framework of health services and care providers are encouraged to identify mental health problems during pregnancy and the postpartum period (16, 17). Therefore, the timely assessment and identification of obsessive-compulsive behaviors during the postpartum period in terms of baby care can help health care professionals and parturient women increase their vigilance toward mental disorders such as obsessive-compulsive disorder, anxiety disorders, and depression, enabling prompt intervention (18).

More than half of patients with obsessive-compulsive disorder have experienced at least one traumatic event, and unexpected accidents or life stressors that involve severe psychological trauma act as precipitating factors for obsessive-compulsive disorder, as a result of which obsessive-compulsive behaviors may develop or escalate in response (19). The 2013 Diagnostic and Statistical Manual of Mental Disorders, Fifth Edition (DSM-5) includes medical-related traumatic stress events such as pregnancy, childbirth, miscarriage, and preterm birth (20). Birth trauma refers to actual or threatened severe harm or even death inflicted on the mother or infant through events or care practices during labor and delivery, and it encompasses both physical and psychological trauma. Pregnancy and childbirth have been recognized as significant risk factors for obsessive-compulsive disorder, and the emergence of postpartum obsessive-compulsive behaviors may help individuals cope with traumatic events or avoid re-experiencing trauma-related symptoms (21). In research on the etiology of obsessive-compulsive disorder outside of the perinatal context (22), an association between trauma exposure and the development of obsessive-compulsive disorder has been identified. On the basis of the above evidence, this study hypothesizes that psychological birth trauma may influence obsessive-compulsive behaviors during the postpartum period in terms of baby care.

The intolerance of uncertainty (IU), which is also referred to as uncertainty intolerance, refers to an individual’s cognitive bias in their perceptions, interpretations, and responses to uncertain situations or events (23). The uncertainty distress model holds that when people face stress or distress, their trait-like intolerance of uncertainty directly affects their cognitive evaluation of the current situation; when cognitive bias occurs toward uncertain events, individuals take a series of behavioral measures to reduce their level of inner uncertainty (24); specifically, parturient women with a low intolerance of uncertainty tend to interpret unknown and uncertain situations as threatening and catastrophic, which triggers intense inner discomfort and anxiety, which causes them to adopt a series of repetitive and controlling behavioral measures to reduce inner uncertainty and alleviate anxiety, which is a behavioral pattern that is highly consistent with the core characteristics of obsessive-compulsive behaviors; therefore, the intolerance of uncertainty can predict obsessive-compulsive tendencies (25). Previous studies have shown that trauma exposure can induce adverse intolerance of uncertainty (26), leading to stronger cognitive biases and negative emotional responses, and the intolerance of uncertainty has been proven to potentially mediate the relationship between trauma and adverse emotional behaviors (27, 28); however, these findings still need to be confirmed through additional research.

According to stress-coping theory, coping is defined as the constantly changing cognitive and behavioral efforts of individuals in their management of specific external or internal demands that are considered to exceed their personal resources, which highlights the importance of coping styles in the process through which stress influences outcomes (29, 30). Studies suggest that joint coping between couples serves as a protective factor against perinatal psychological problems (31, 32), but that parturient women with obsessive-compulsive disorder may experience more marital distress and less social support (33). Dyadic coping is a specific coping pattern that refers to the responses and shared decision-making of both partners in the face of stressful events, where the stress and coping processes that the spouses undertake together is inherently interrelated, which means that such partners tend to respond as a unit rather than as individuals when confronted with uncertain events (34). Research conducted outside of the perinatal context has shown that adaptive coping styles can negatively predict obsessive-compulsive tendencies (35), and coping styles have been frequently demonstrated to mediate the relationship between traumatic experiences and mental health (36). Therefore, the mechanism of dyadic coping warrants in-depth analysis in the exploration of stress-related psychological phenomena such as obsessive-compulsive behaviors during the postpartum period in terms of baby care.

In view of the relationships among these variables, we hypothesize that psychological birth trauma may not only directly affect mothers’ obsessive-compulsive behaviors during the postpartum period in terms of baby care but also indirectly influence such behaviors through intolerance of uncertainty and dyadic coping. To date, population-based data concerning baby care among Chinese parturient women who exhibit obsessive-compulsive behaviors during the postpartum period are lacking, and whether psychological birth trauma, the intolerance of uncertainty, and dyadic coping affect and mediate this process remains unclear. Given this research gap, we designed a cross-sectional survey to explore the interrelationships and mechanisms underlying psychological birth trauma, the intolerance of uncertainty, dyadic coping, and obsessive-compulsive behaviors during the postpartum period with respect to baby care. We propose the following hypotheses: (a) Postpartum women exhibit obsessive-compulsive behavioral symptoms in terms of infant care; (b) women with higher levels of psychological birth trauma are more likely to display higher levels of obsessive-compulsive behaviors in baby care during the postpartum period; and (c) intolerance of uncertainty and dyadic coping play serial mediating roles between psychological birth trauma and obsessive-compulsive behaviors in terms of baby care during the postpartum period.

## 2 Materials and methods

### 2.1 Study design and procedure

In this study, a longitudinal design was employed. Data were collected at two time points: the first is at 1–3 days post-partum or upon hospital discharge (T1), during which general demographic information, psychological birth trauma, and intolerance of uncertainty were assessed; at 1 month post-partum (T2) (4), dyadic coping and obsessive-compulsive behaviors in baby care during the postpartum period were measured. Prior to the survey, the researchers fully explained the study purpose, completion instructions, and privacy protection measures to the participants, and the questionnaires were administered only after informed consent was obtained. The questionnaires were completed and collected offline, with the researchers providing timely assistance to ensure a valid response rate. For the second data collection phase, the participants were contacted via telephone or short message services and asked to complete the questionnaires online.

This study protocol was approved by the Ethics Review Committee of Shandong Provincial Hospital Affiliated with Shandong First Medical University (SWYX: NO.2025–721).

### 2.2 Participants

The sample size was calculated using G*Power, yielding a minimum required sample of 198; anticipating a 20% attrition rate during data collection, at least 248 participants were targeted for recruitment. A convenience sampling method was used to enroll postpartum women who gave birth to singletons and were hospitalized at a tertiary hospital in Shandong Province between October 2025 and February 2026, with eligibility criteria including being older than18 years and having provided informed voluntary consent. The exclusion criteria were severe postpartum complications that required transfer to other departments, persistent mother–infant separation during the investigation period, neonatal death after delivery, major nonpregnancy- and childbirth-related traumatic events having occurred in the past month (e.g., traffic accidents, natural disasters, or bereavement), and cognitive or hearing impairments that hindered accurate understanding of the questionnaire items.

### 2.3 Measures

#### 2.3.1 General information questionnaire

General maternal data included age, marital status, employment status, educational level, monthly household income, parity, conception method, mode of delivery, preterm birth status, gestational complications, history of abortion or stillbirth, and unintended pregnancy status. General neonatal data included sex, birth weight, primary caregiver, breastfeeding pattern, history of admission to the neonatal intensive care unit (NICU), and the presence of birth defects.

#### 2.3.2 Psychological birth trauma scale

In 2024, the Chinese researcher Sun et al. (37) developed this scale on the basis of a conceptual analysis of psychological birth trauma (PBT). The PBT Scale comprises two components, namely, intrapartum experiences and postpartum effects, and encompasses six dimensions, namely, lack of support, loss of control, dignity deprivation, threat to safety, physical and psychological symptoms, and negative cognition. The scale has a total of 29 items rated on a 5-point Likert scale (1 = strongly disagree, 2 = disagree, 3 = neutral, 4 = agree, and 5 = strongly agree), among which Items 1, 2, 4, 10, and 16 are reverse scored. Postpartum women completed self-assessments according to their actual experiences; higher total scores indicate more severe PBT. The scale is applicable to both vaginal and cesarean delivery and enables a comprehensive evaluation of PBT severity. In the present study, the Cronbach’s *α* coefficient of this scale was 0.897.

#### 2.3.3 Chinese version of the intolerance of uncertainty Scale-12 (IUS-12)

The original French version of the Intolerance of Uncertainty Scale was developed by Freeston et al. and consists of 27 items across five factors. In 2002, Buhr et al. (38) adapted the scale into English, and in 2007, Carleton et al. (39) developed a 12-item short form of the IUS. In 2016, the Chinese scholar Li-juan Wu et al. (23) translated and validated the short-form IUS into Chinese, which includes 12 items covering three dimensions: prospective anxiety (6 items), inhibitory behavior (3 items), and prospective emotion (3 items). A 5-point scoring system (1 = strongly inconsistent, 2 = inconsistent, 3 = uncertain, 4 = consistent, and 5 = strongly consistent) is used for this scale, with higher scores representing greater levels of uncertainty tolerance. In the present study, the Cronbach’s *α* coefficient of the total Chinese IUS-12 scale was 0.843.

#### 2.3.4 Dyadic coping inventory (DCI)

The DCI was developed by Bodenmann et al. and translated into Chinese by Xu et al. (40) to assess the dyadic coping capacity across patients and their spouses. It contains six dimensions: stress communication (8 items), supportive dyadic coping (10 items), common dyadic coping (5 items), delegated dyadic coping (4 items), negative dyadic coping (8 items), and coping quality evaluation (2 items). A 5-point Likert scale is used, with responses ranging from 1 (rarely) to 5 (very frequently). The items for measuring negative dyadic coping are reverse scored, and the two items in the quality evaluation dimension are not included in the total score. The total score ranges from 35 to 175, with higher scores indicating better positive dyadic coping; scores below 111 indicate below-average levels, scores from 111 to 145 indicate average levels, and scores above 145 indicate above-average levels. The Cronbach’s *α* coefficient of this scale in the present study was 0.935.

#### 2.3.5 Obsessive and compulsive behaviors scale for mothers in the postpartum period in terms of baby care (OCBS-MPPRBC)

Developed in English in 2019 by Özdemir et al. (41) and translated into Chinese in 2025 by Chinese scholars (4), this scale is used to assess obsessive-compulsive symptoms related to infant care among mothers within the 2–8 weeks post-partum period. It consists of 9 items across 2 dimensions, responses are rated on a 5-point Likert scale, and total scores range from 9 to 45 (sum of all items); higher scores indicate more severe obsessive-compulsive behaviors during the postpartum period in terms of baby care. The Cronbach’s *α* coefficient of the scale in this study was 0.829.

### 2.4 Statistical analysis

Eight cases (3.1, <5%) of missing data regarding household income were imputed via the mode imputation method (42). Data cleaning and statistical analyses were performed using the PROCESS macro for SPSS 26.0 (43). A significance level of *α* = 0.05 was adopted, with *p* < 0.05 considered to indicate statistical significance. Given the questionnaire-based data collection, potential common method bias was assessed using Harman’s single-factor test; the first factor accounted for less than 40% of the total variance, indicating that there was no severe common method bias in the dataset. Categorical data are presented as frequencies and percentages, and normally distributed continuous data are presented as the mean ± standard deviation. Independent-samples *t*-tests and one-way analysis of variance (ANOVA) were used to examine the effects of sociodemographic characteristics on obsessive-compulsive behaviors during the postpartum period in terms of baby care. A fetus is considered to be preterm if it is born before 37 completed weeks of gestation (44). All the data were approximately normally distributed; Pearson correlation analysis was therefore performed to examine the bivariate correlations among psychological birth trauma, intolerance of uncertainty, dyadic coping, and obsessive-compulsive behaviors during the postpartum period in terms of baby care. Multiple linear regression was conducted to identify factors influencing obsessive-compulsive behaviors during the postpartum period in terms of baby care. Continuous variables were standardized, and factors showing significant effects in the regression analysis were included as confounders. Serial mediation analysis was performed using Model 6 in the PROCESS macro for SPSS 26.0, and the bootstrap method (5,000 resamples) was used to test mediation effects; a 95% confidence interval not containing 0 indicates a significant mediation effect.

## 3 Results

### 3.1 Common method bias test

The common method bias test results revealed that for the data on psychological birth trauma and intolerance of uncertainty that had been collected at T1, there were 10 factors with eigenvalues greater than 1, with the first factor accounted for 21.846% of the total variance; for data on dyadic coping and obsessive-compulsive behaviors in terms of baby care during the postpartum period were collected at T2, and 10 factors had eigenvalues greater than 1, with the first factor explaining 27.338% of the total variance, which indicates that there was no severe common method bias in this study.

### 3.2 Sociodemographic characteristics

In the first phase of the study, a total of 317 participants were recruited, yielding 309 completed questionnaires; after the exclusion of invalid and monotonous responses, 305 valid first-phase questionnaires were retained. During the second-phase survey, 2 parturient women were still in mother–infant separation, 18 could not be contacted for various reasons, and 7 had withdrawn from the study, resulting in a final sample of 278 parturient women included in the analysis, for an attrition rate of 8.85%. A history of previous psychiatric disorders was also collected, and all of the enrolled parturient women reported no history of related mental disorders. The baseline demographic and clinical characteristics of the participants are presented in Table 1.

**Table 1:** Demographic characteristics and univariate analysis results of OCBS-MPPRBC.

Variable	Group	Number	Percentage %	OCBS-MPPRBC	*t*/*F*	*p*
Mothers						
Age (years)	<30	104	37.4	20.9 ± 6.020	2.247	0.108
	30 ~ 34	88	31.7	22.05 ± 6.072		
	≥35	86	30.9	20.05 ± 6.677		
Marital status	Married	272	97.8	21.01 ± 6.337	0.735	0.485
	Unmarried	6	2.2	20.33 ± 2.066		
Employment status	Work-from-home	20	7.2	19.15 ± 5.412	1.135	0.323
	On maternity leave	217	78.1	21.04 ± 6.069		
	Unemployed	41	14.7	21.71 ± 7.590		
Educational level	High school or below	24	8.6	22.46 ± 7.774	0.711	0.492
	College/bachelor’s degree	205	73.7	20.88 ± 6.253		
	Postgraduate or above	49	17.6	20.80 ± 5.560		
Monthly household income (yuan)	<5,000	18	6.5	25.67 ± 6.544	10.326	<0.001**
	5,000 ~ 8,000	92	33.1	22.23 ± 6.498		
	>8,000	168	60.4	19.83 ± 5.777		
Parity	Parity	173	62.2	22.50 ± 5.903	5.355	<0.001**
	Multiparous	105	37.8	18.53 ± 6.113		
Unintended pregnancy	Yes	56	20.1	21.80 ± 5.735	1.073	0.284
	No	222	79.9	20.80 ± 6.400		
Conception method	Natural conception	252	90.6	20.46 ± 6.045	−4.589	<0.001**
	Assisted reproductive technology	26	9.4	26.19 ± 6.197		
Mode of delivery	Vaginal delivery	138	49.6	20.63 ± 6.029	−0.975	0.33
	Cesarean section	140	50.4	21.36 ± 6.509		
Preterm birth	Yes	36	12.9	24.08 ± 5.784	3.213	0.001**
	No	242	87.1	20.54 ± 6.226		
Gestational complications	Yes	170	61.2	21.78 ± 6.463	2.616	0.009**
	No	108	38.8	19.78 ± 5.786		
History of abortion	Yes	103	37.1	20.37 ± 6.039	−1.288	0.199
	No	175	62.9	21.37 ± 6.397		
Neonates						
Primary infant caregiver after discharge	Infant care institution	114	41	20.04 ± 6.069	3.862	0.01*
	Mother	89	32	22.82 ± 6.130		
	Father	22	7.9	20.73 ± 6.378		
	Other family members	53	19.1	20.11 ± 6.414		
Breastfeeding pattern	Exclusive breastfeeding	64	23	20.72 ± 6.025	0.087	0.916
	Mixed feeding	205	73.7	21.09 ± 6.340		
	Formula feeding	9	3.2	20.89 ± 7.184		
Infant gender	Male	146	52.5	20.68 ± 6.437	−0.899	0.369
	Female	132	47.5	21.36 ± 6.095		
Birth weight (g)	<2,500	18	6.5	25.72 ± 6.201	6.307	0.002**
	2,500 ~ 4,000	250	89.9	20.76 ± 6.119		
	≥4,000	10	3.6	18.50 ± 7.075		
History of NICU admission	Yes	45	16.2	25.09 ± 6.003	4.975	<0.001**
	No	233	83.8	20.21 ± 6.025		
Birth defects	Yes	20	7.2	24.95 ± 5.453	2.963	0.003**
	No	258	92.8	20.69 ± 6.239		

### 3.3 Status and influencing factors of obsessive-compulsive behaviors during the postpartum period in terms of baby care

Among the 278 postpartum women in this study, the OCBS-MPPRBC total scores ranged from 9 to 39, with a mean score of 21 ± 6.275 (moderate-to-low level); the obsessive-compulsive behavior subscale scores ranged from 4 to 18, with a mean of 9.130 ± 3.105, and the obsessive-compulsive cognition subscale scores ranged from 5 to 24, with a mean of 11.8705 ± 4.100, which indicate a slightly higher score in the obsessive-compulsive cognition dimension. Univariate analysis revealed significant differences in the total scores for obsessive-compulsive behaviors during the postpartum period in terms of baby care across monthly household income (*F* = 10.326, *p* < 0.001), parity (*t* = 5.355, *p* < 0.001), conception method (*t* = −4.589, *p* < 0.001), preterm birth (*t* = 3.213, *p* = 0.001), gestational complications (*t* = 2.616, *p* = 0.009), primary infant caregiver after discharge (*F* = 3.862, *p* = 0.01), birth weight (*F* = 6.307, *p* = 0.002), history of NICU admission (*t* = 4.975, *p* < 0.001), and birth defects (*t* = 2.963, *p* = 0.003), as detailed in Table 1. Multiple linear regression was conducted on obsessive-compulsive behaviors during the postpartum period, with baby care used as the dependent variable and statistically significant factors from univariate analysis set as independent variables after coding the categorical variables, as shown in Supplementary Table 1. The tolerance values of the independent variables ranged from 0.775 to 0.967 (> 0.1), and the variance inflation factors ranged from 1.034 to 1.09 (< 10), suggesting that no severe multicollinearity occurred among the variables. Monthly household income (*β* = 0.101, *p* = 0.023), parity (*β* = 0.242, *p* < 0.001), conception method (*β* = −0.151, *p* = 0.001), primary infant caregiver (*β* = 0.158, *p* < 0.001), history of NICU admission (*β* = 0.192, *p* < 0.001), psychological birth trauma (*β* = 0.108, *p* = 0.029), intolerance of uncertainty (*β* = 0.411, *p* < 0.001), and dyadic coping (*β* = −0.187, *p* < 0.001) were entered into the regression equation, which collectively explained 47.9% of the total variance in obsessive-compulsive behaviors during the postpartum period in terms of baby care, as presented in Table 2.

**Table 2:** Results of multiple linear regression analysis for influencing factors of obsessive-compulsive behaviors during postpartum infant care.

						Collinearity diagnostics	
Variable	Unstandardized regression (*B*)	Standard error (*SE*)	Standardized regression coefficient (*β*)	t statistic (*t*)	*P*-value	Tolerance	Variance inflation factor (VIF)
(Constant)	18.145	2.898		6.261	<0.001		
Intolerance of Uncertainty	0.379	0.045	0.411	8.434	<0.001	0.786	1.272
NICU	3.269	0.758	0.192	4.315	<0.001	0.943	1.061
Parity	3.125	0.585	0.242	5.338	<0.001	0.911	1.097
Dyadic Coping	−0.076	0.019	−0.187	−4.111	<0.001	0.908	1.101
primary infant caregiver	2.117	0.594	0.158	3.564	<0.001	0.956	1.046
natural conception	−3.243	0.963	−0.151	−3.369	0.001	0.934	1.07
Monthly household income (yuan)							
<5,000	2.564	1.12	0.101	2.29	0.023	0.967	1.034
5,000 ~ 8,000	–	–	–	–	–	–	–
Psychological birth trauma	0.05	0.023	0.108	2.198	0.029	0.775	1.290

### 3.4 Bivariate correlation analysis

The results presented in Table 3 indicate that the maternal OCBS-MPPRBC scores were positively correlated with psychological birth trauma (*r* = 0.373, *p* < 0.01) and intolerance of uncertainty (*r* = 0.493, *p* < 0.01) but negatively correlated with dyadic coping (*r* = −0.177, *p* < 0.01), psychological birth trauma was positively associated with intolerance of uncertainty (*r* = 0.400, *p* < 0.01) and negatively associated with dyadic coping (*r* = −0.126, *p* < 0.05), and intolerance of uncertainty was positively correlated with dyadic coping (*r* = 0.140, *p* < 0.05).

**Table 3:** Overall correlation analysis among variables.

Variable	Psychological birth trauma	Intolerance of uncertainty	Dyadic coping	OCBS-MPPRBC	M ± SD
Psychological birth trauma	1				59.06 ± 13.619
Intolerance of uncertainty	0.400**	1			25.04 ± 6.816
Dyadic coping	−0.126*	0.140*	1		130.32 ± 15.342
OCBS-MPPRBC	0.373**	0.493**	−0.177**	1	21.00 ± 6.275

### 3.5 Serial mediation effect analysis

**Figure 1:**
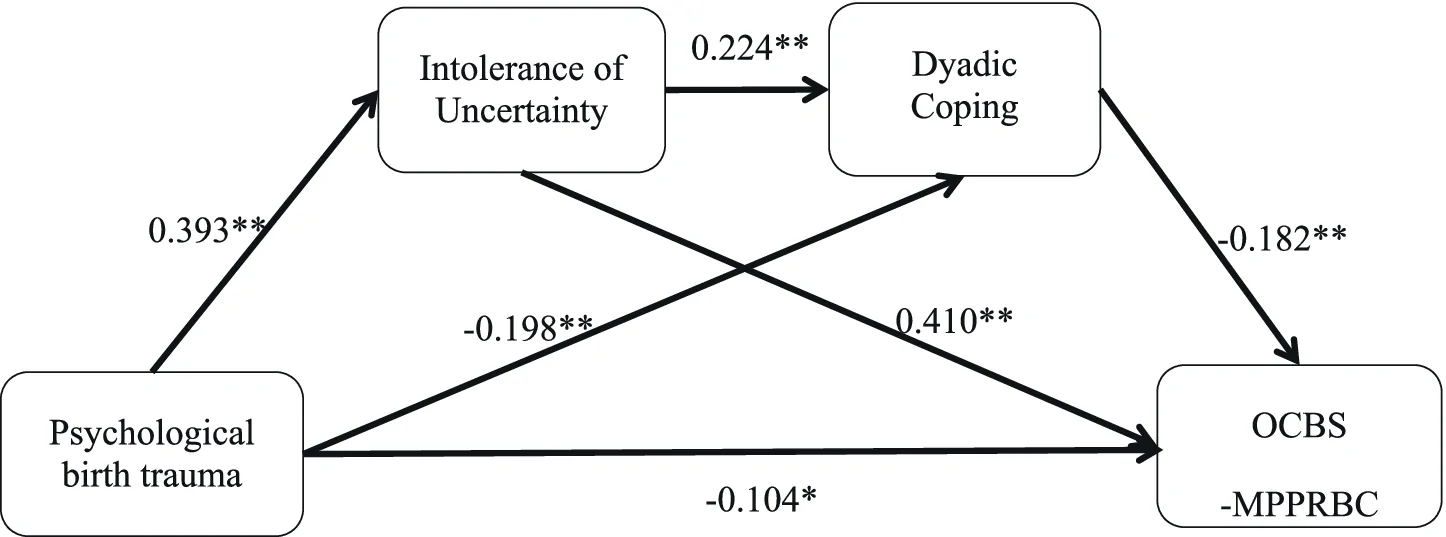
Schematic diagram of the serial mediation effect.

In this study, standardized psychological birth trauma was used as the independent variable, standardized obsessive-compulsive behaviors during the postpartum period in terms of baby care were used as the dependent variable, and standardized intolerance of uncertainty and dyadic coping were used as the mediating variables, with sociodemographic variables, which include monthly household income, parity, conception method, primary infant caregiver after discharge, and history of NICU admission, controlled to minimize confounding effects and improve the explanatory power of the model. As shown in Table 4, psychological birth trauma had a significant positive effect on intolerance of uncertainty (*β* = 0.393, *p* < 0.001), indicating that higher levels of psychological birth trauma were associated with greater levels of intolerance of uncertainty; psychological birth trauma had a significant negative effect on dyadic coping (*β* = −0.198, *p* < 0.01), indicating that higher levels of psychological birth trauma were associated with poorer dyadic coping; intolerance of uncertainty had a significant positive effect on dyadic coping (*β* = 0.224, *p* < 0.001), suggesting that higher levels of intolerance of uncertainty were related to higher levels of dyadic coping; psychological birth trauma had a significant positive effect on OCBS-MPPRBC scores (*β* = 0.104, *p* < 0.05), indicating that higher levels of psychological birth trauma corresponded to higher OCBS-MPPRBC scores; intolerance of uncertainty was positively associated with OCBS-MPPRBC scores (*β* = 0.410, *p* < 0.001), indicating that a higher level of intolerance of uncertainty corresponded to greater severity of obsessive-compulsive symptoms; and dyadic coping was negatively associated with OCBS-MPPRBC scores (*β* = −0.182, *p* < 0.001), suggesting that better dyadic coping relates to lower OCBS-MPPRBC scores. Further bootstrap testing revealed a weak serial mediating effect of uncertainty tolerance and dyadic coping between psychological birth trauma and OCBS-MPPRBC, with an effect value of 0.181 and a total indirect effect ratio of 63.38%. Specifically, three significant mediating paths were identified: path 1 (psychological birth trauma → intolerance of uncertainty → OCBS-MPPRBC), with an effect value of 0.161 (56.4%), path 2 (psychological birth trauma → Dyadic coping → obsessive-compulsive behaviors), with 0.036 (12.59%), and path 3 (psychological birth trauma → intolerance of uncertainty → dyadic coping → obsessive-compulsive infant care behaviors), with an effect value of −0.016 (−5.61%), All of which were statistically significant but yielded small to trivial effect sizes, indicating limited practical clinical implications, as detailed in Table 5 and Figure 1.

**Table 4:** Serial mediation analysis of intolerance of uncertainty and dyadic coping in the relationship between psychological birth trauma and OCBS-MPPRBC.

					95%CI			
Outcome variable (dependent variable of each regression model)	Predictor (independent predictive variable)	Standardized regression coefficient (*β*)	Standard error (*SE*)	t-test statistic(*t*)	Lower limit of 95% confidence interval (LLCI)	upper limit of 95% confidence interval (ULCI)	Coefficient of determination (*R*^2^)	F statistic for overall model significance (*F*)
Intolerance of uncertainty	Psychological birth trauma	0.393	0.057	6.898	0.280	0.505	0.178	8.374
Dyadic coping	Psychological birth trauma	−0.198	0.065	−3.052	−0.32	−0.070	0.098	3.662
	Intolerance of uncertainty	0.224	0.064	3.518	0.099	0.350		
OCBS-MPPRBC	Psychological birth trauma	0.104	0.049	2.124	0.008	0.201	0.500	29.736
	Intolerance of Uncertainty	0.410	0.049	8.404	0.314	0.506		
	Dyadic coping	−0.182	0.046	−3.996	−0.271	−0.092		

**Table 5:** Serial mediating effects of intolerance of uncertainty and dyadic coping between psychological birth trauma and OCBS-MPPRBC.

Mediation effect path	Effect value	SE	95%CI	Effect ratio (%)
Total effect	0.285	0.050	0.186–0.384	
Direct effect	0.104	0.049	0.008–0.201	36.62
Total indirect effect	0.181	0.029	0.126–0.240	63.38
Psychological birth trauma-intolerance of Uncertainty- OCBS-MPPRBC	0.161	0.026	0.111–0.214	56.40
Psychological birth trauma–dyadic coping–OCBS-MPPRBC	0.036	0.016	0.009–0.070	12.59
Psychological birth trauma–intolerance of uncertainty–dyadic coping–OCBS-MPPRBC	−0.016	0.007	−0.031–0.004	−5.61

## 4 Discussion

This study confirmed that psychological birth trauma serves as a critical risk factor for postpartum obsessive-compulsive behaviors, and verified the serial mediating effects of intolerance of uncertainty and dyadic coping. The findings support the core propositions of existing perinatal psychological theories regarding the fundamental assumption of intolerance of uncertainty. Meanwhile, the protective and buffering effect of dyadic coping is consistent with the basic tenet of stress-coping theory that positive interpersonal coping can mitigate adverse psychological outcomes.

In this study, the mean OCBS-MPPRBC score of 278 parturients was 21.00 ± 6.275, suggesting a relatively low level (45, 46); the mean score of the obsessive-compulsive cognition dimension was slightly higher than that of the obsessive-compulsive behavior dimension. Compared with other studies that used the same scale (12, 47), the OCBS-MPPRBC scores in this study were slightly lower, which can be attributed to the fact that these prior studies were conducted during the COVID-19 pandemic, during which maternal fear of the pandemic potentially exacerbated obsessive-compulsive behaviors (45, 48).

The results of the multivariate analysis are consistent with findings from several perinatal mental health studies (12, 47, 49, 50). Compared with multiparous women, primiparous women had significantly higher OCBS-MPPRBC scores. Primiparas lack experience in infant care and are therefore more sensitive to various uncertainties during caregiving (51, 52), whereas multiparas have accumulated rich caregiving experience, are more familiar with infants’ normal physiological responses and care routines, present lower anxiety levels, and, correspondingly, engage in fewer obsessive-compulsive behaviors. Although the sample of women who conceived via assisted reproductive technology was small in this study, significant postpartum infant care-related obsessive-compulsive behaviors were still exhibited among this group. A possible explanation is that the women in this group had undergone a longer period of preconception preparation and medical treatment, which led to stronger emotional attachment to their infants and a heightened perception of health risks, which can result in greater parenting difficulties and higher levels of anxiety regarding infant survival, thereby predisposing the women in this group to engage in overprotective caregiving behaviors (50). A significant association between *in vitro* fertilization and postpartum negative emotions or symptoms were not detected in other inconsistent perinatal mental health studies, which may be attributed to factors such as distinct treatment protocols and psychological resilience (53–55). In addition, preterm birth is considered to be an important predictor of postpartum obsessive-compulsive disorder, whereas our multivariate analysis revealed no significant difference, which can be explained by the small number of preterm infants sampled in this study, most of whom were late preterm infants with no significant differences from term infants (12); in contrast, parents of early preterm infants may be more prone to obsessive-compulsive behaviors. It is well acknowledged that postpartum compulsive behaviors stem from multiple factors. Low income, primiparity, conception via assisted reproductive technology and neonatal NICU admission may exert cumulative effects, amplifying the adverse impacts of psychological birth trauma and exacerbating cognitive biases and parenting anxiety. By contrast, higher income and multiparity can serve as protective factors through resource advantages and accumulated experience, reducing the risk of such behaviors. These findings call for a dialectical interpretation of the research results.

The results revealed that birth-related psychological trauma had a significant direct positive effect on postpartum infant care-related obsessive-compulsive behaviors and that higher levels of birth-related psychological trauma were associated with more severe obsessive-compulsive caregiving behaviors among postpartum women, which confirms the research hypothesis positing that birth trauma serves as a risk factor for perinatal obsessive-compulsive disorder. This is consistent with previous findings regarding childhood trauma (22, 56), whereas other factors, such as family dysfunction and family disharmony, may have serve as potential traumatic sources. Dysfunction of the cortico-striatal-thalamic-cortical (CSTC) circuit constitutes the core neuropathological mechanism of obsessive-compulsive disorder. Childbirth trauma elevates cortisol levels and disrupts prefrontal-striatal neural regulation, further impairing CSTC circuit balance and undermining cognitive control and emotional modulation. Such circuit dysregulation triggers excessive risk vigilance and reduced behavioral inhibition, thereby inducing compulsive behaviors like repetitive checking and excessive cleaning. A previous study (46) suggested that discomfort during pregnancy can affect childbirth-related anxiety and emotional changes and that obsessive-compulsive behaviors can emerge when women fail to cope effectively. Similarly, physical and mental symptoms and negative cognitions triggered by birth-related psychological trauma can impair maternal psychological adjustment, making the adaptation to postpartum role transition and caregiving stress more difficult (47).

The present study reveals that intolerance of uncertainty exerted a significant positive mediating effect between psychological birth trauma and postpartum infant care-related obsessive-compulsive behaviors. Although this relationship had been directly explored in few studies, relevant adolescent studies (27, 28) have confirmed the core role of the intolerance of uncertainty in the association between trauma and negative emotions or psychiatric disorders. Trauma exposure can lead individuals to develop a belief system that positions uncertainty as inherently dangerous, which undermines maternal psychological security; parturient women with high levels of intolerance of uncertainty experience chronic anticipatory anxiety and hypervigilance and adopt behavioral avoidance toward uncertain stimuli and situations, thereby exacerbating their care-related obsessive-compulsive behaviors (27). This high contribution of the mediating pathway indicates that the effect of birth trauma on postpartum infant care-related obsessive-compulsive behaviors is predominantly an indirect transmission process centered on cognitive biases. As a stable vulnerability trait, intolerance of uncertainty plays a fundamental and dominant regulatory role in the development of post-traumatic behavioral abnormalities, surpassing superficial mediators such as emotion and stress. This finding explains why some women with mild childbirth trauma still experience persistent obsessive care behaviors, offering a novel cognitive target for targeted interventions on postpartum psychological and behavioral problems.

Dyadic coping also significantly positively mediated the relationship between psychological birth trauma and postpartum infant care-related obsessive-compulsive behaviors. The results of the present study indicate that psychological birth trauma was significantly negatively correlated with dyadic coping, revealing that higher levels of psychological birth trauma, the poorer maternal dyadic coping capacity and the more severe postpartum care-related obsessive-compulsive behaviors (36, 57). Postpartum women who experience birth trauma may exhibit negative psychological states, and emotional exhaustion can impair their adaptation to postpartum self-care and infant care. In addition, the support of husbands or family members is critical for the alleviation of negative coping styles, yet some women report insufficient support from their spouses and relatives (58). However, other studies have suggested that the majority of postpartum women do receive adequate support from husbands, family, or society, which buffers the negative effects of adverse delivery or postpartum recovery experiences (59, 60). Such inconsistencies may stem from variations in cultural and family backgrounds, as well as maternal personality traits and other potential variables, including heightened fear of childbirth, poor maternal role adaptation, low self-efficacy, and burnout (12, 45), which should be included in future studies for further analysis.

In this study, the chain mediating role of intolerance of uncertainty and dyadic coping was verified, indicating that while intolerance of uncertainty intensifies maternal obsessive-compulsive caregiving behaviors, it can also indirectly alleviate such behaviors by enhancing the capacity for dyadic coping. The results reveal a significant positive correlation between intolerance of uncertainty and dyadic coping. Contrary to conventional findings, intolerance of uncertainty may not merely represent a pathological negative trait. Under the high-stress context of postpartum infant care and shaped by indigenous Chinese cultural norms, postpartum women tend not to endure parenting pressure alone. Persistent care-related anxiety prompts mothers to convey stress signals to their partners and share childcare burdens collaboratively (61). Long-established family support dynamics effectively weaken the avoidant tendency associated with high intolerance of uncertainty (62, 63). In addition, potential methodological limitations inherent in self-reported measurements may induce spurious positive correlations and overestimate the actual association between the two variables. More rigorous research designs are warranted in future studies. These findings provide novel insights for targeted clinical intervention. On the one hand, a multidisciplinary team consisting of obstetricians, pediatricians, and psychological specialists should be established to target high-risk parturient women with severe psychological birth trauma and elevated intolerance of uncertainty (64). Psychological professionals can deliver individualized cognitive behavioral interventions, provide professional parenting education prior to hospital discharge, and conduct postpartum follow-ups via video, telephone, or intelligent health management platforms to dynamically evaluate maternal psychological status and infant care behaviors, and relieve their confusion regarding childcare. On the other hand, perinatal health education courses should be provided for couples jointly to guide partners to actively engage in infant care, buffer the stress induced by childbirth trauma and parenting challenges through family support, and effectively reduce the risk of postpartum infant care-related obsessive-compulsive behaviors.

This study extends the application of intolerance of uncertainty and stress-coping theories to the field of perinatal mental health, constructs a serial mediating theoretical framework linking psychological birth trauma to postpartum infant care-related obsessive-compulsive behaviors, providing empirical evidence for early screening, risk stratification, and targeted intervention among perinatal women at high psychological risk; however, several limitations remain in the present research. First, this study only recruited postpartum women from urban tertiary hospitals and did not include samples from primary medical institutions and remote areas. Inherent heterogeneity in educational attainment and household economic status exists across different maternal groups. Differences in obstetric service models and service standards between tertiary and grassroots hospitals further constrain result extrapolation. Meanwhile, distinct social cultures and parenting beliefs between China and Western countries also limit the cross-cultural generalizability of the findings to a certain extent. Second, postpartum sleep deprivation and poor sleep quality are common physiological and psychological stressors among perinatal women, which readily induce negative mental problems such as postpartum anxiety and depression. Perinatal obsessive-compulsive disorder exhibits high comorbidity with anxiety and depression, while adequate social support can effectively reduce the risk of adverse mental health outcomes. Therefore, future research should further incorporate classic perinatal psychological variables, including sleep quality, social support, previous psychological distress, postpartum anxiety and depression, to systematically explore the formation mechanism of infant care-related obsessive-compulsive behaviors under the interaction of multiple factors, and provide comprehensive theoretical evidence and empirical support for establishing a multidimensional perinatal psychological screening and intervention system. Third, the longitudinal follow-up period was relatively short, and there were only two waves of data collection, while postpartum obsessive-compulsive symptoms may progress or remit within 6 months post-partum; thus, the dynamic changes in postpartum infant care-related obsessive-compulsive behaviors were not analyzed through longer-term tracking.

## 5 Conclusion

This study explored the relationships between psychological birth trauma, intolerance of uncertainty, dyadic coping, and postpartum infant care-related obsessive-compulsive behaviors, as well as their serial mediating effects. It clarifies the underlying mechanisms of postpartum obsessive-compulsive behaviors, expands the application of stress-coping theory in perinatal mental health, suggesting that intolerance of uncertainty may possess certain adaptive functions. The findings provide evidence for early screening and couple-oriented psychological intervention. Future multi-center longitudinal studies are required to verify model stability and develop targeted preventive strategies.

## Data Availability

The raw data supporting the conclusions of this article will be made available by the authors, without undue reservation.
