# Correction: Effects of psychological birth trauma on obsessive-compulsive behaviors during the postpartum period in terms of infant care: the chain-mediating role of intolerance of uncertainty and dyadic coping

**DOI:** 10.3389/fpubh.2026.1952843

**Published:** 2026-08-18

**Authors:** Xiaotong Zhang, Qi Xu, Minmin Li, Ye Zhang, Jia Song, Xia Liu

**Affiliations:** 1 School of Nursing, Shandong First Medical University, Taian, China; 2 Department of Neonatology, East Campus, Shandong Provincial Hospital Affiliated to Shandong First Medical University, Jinan, China; 3 Department of Obstetrics, East Campus, Shandong Provincial Hospital Affiliated to Shandong First Medical University, Jinan, China; 4 Department of Nursing, Shandong Provincial Hospital Affiliated to Shandong First Medical University, Jinan, China

**Keywords:** birth trauma, chain mediating effects, infant care, obsessive-compulsive behaviors, obsessive-compulsive disorder

An incorrect **Funding** statement was provided. The correct **Funding** statement reads:

The author(s) declared that financial support was received for this work and/or its publication. This work was supported by the Horizontal Project of Shandong Provincial Hospital Affiliated to Shandong First Medical University (Grant No. 2023370104147030).

The original version of this article has been updated.

