# Supplementary material for: Effects of psychological birth trauma on obsessive-compulsive behaviors during the postpartum period in terms of infant care: the chain-mediating role of intolerance of uncertainty and dyadic coping
Source: Front Public Health. 2026 Jul 15;14:1876448. doi: 10.3389/fpubh.2026.1876448 (PMC13414954; doi:10.3389/fpubh.2026.1876448)
Supplement: Supplementary file 1 [file Table_1.DOCX]

Supplementary Material

**Supplementary Table 1.** Coding of Independent Variables and Dummy Variables for Multiple Linear Regression of Postpartum Obsessive-Compulsive Behaviors

| Variable | Dummy Variable | Coding |
| --- | --- | --- |
| Monthly household income (yuan) | ＞8000 | （reference） |
|  | 5000～8000 | 5000～8000=1，＜5000=0，＞8000=0 |
|  | ＜5000 | ＜5000=1，5000～8000=0，＞8000=0 |
| Conception method | assisted reproductive technology | （reference） |
|  | natural conception | natural conception =1，  assisted reproductive technology =0 |
| Preterm birth | No | （reference） |
|  | Yes | Yes =1，No =0 |
| Gestational complications | No | （reference） |
|  | Yes | Yes =1，No =0 |
| Birth weight (g) | ≥4000 | （reference） |
|  | 2500～4000 | 2500～4000=1，＜2500=0，≥4000=0 |
|  | ＜2500 | ＜2500=1，2500～4000=0，≥4000=0 |
| NICU admission | No | （reference） |
|  | Yes | Yes =1，No =0 |
| Birth defects | No | （reference） |
|  | Yes | Yes =1，No =0 |
| primary infant caregiver after discharge | Mother | （reference） |
|  | other family members or maternity | other family members or maternity =1，  Mother =0 |
| Psychological birth trauma | - | raw scores |
| Intolerance of Uncertainty | - | raw scores |
| Dyadic Coping | - | raw scores |
